# The impacts of nicotinamide and inositol on the available cells and product performance of industrial baker's yeasts

**DOI:** 10.1186/s40643-023-00661-4

**Published:** 2023-07-22

**Authors:** Chengpeng Shan, Tianqing Xia, Jiao Liu, Ying Wang, Penggang Bai, Lili Xu, Zailu Li, Jianzhi Zhao, Xiaoming Bao

**Affiliations:** 1 State Key Laboratory of Biobased Material and Green Papermaking, School of Bioengineering, Qilu University of Technology (Shandong Academy of Sciences), Jinan, 250353 Shandong People’s Republic of China; 2 Shandong Shouguang Juneng Golden Corn Co., Ltd, Shouguang, 262711 Shandong People’s Republic of China

**Keywords:** Baker’s yeast, Nicotinamide, Inositol, Leavening performance, Trehalose synthesis

## Abstract

A suitable nutrient supply, especially of vitamins, is very significant for the deep display of the inherent genetic properties of microorganisms. Here, using the chemically defined minimal medium (MM) for yeast, nicotinamide and inositol were confirmed to be more beneficial for the performance of two industrial baker's yeasts, a conventional and a high-sugar-tolerant strain. Increasing nicotinamide or inositol to proper levels could enhance the both strains on cell growth and activity and product performance, including trehalose accumulation and leavening performance. The activity of key enzymes (PCK, TPS) and the content of intermediate metabolites (G6P, UDPG) in the trehalose synthesis pathway were promoted by a moderate supply of nicotinamide and inositol. That were also proved that an appropriate amount of niacinamide promoted the transcription of longevity-related genes (*PNC1*, *SIR2*), and the proper concentration of inositol altered the phospholipid composition in cells, namely, phosphatidylinositol and phosphatidyl choline. Furthermore, the cell growth and the leavening performance of the both strains were promoted after adjusting inositol to choline to the proper ratio, resulting directly in content changes of phosphatidylinositol and phosphatidyl choline in the cells. While the two strains responded to the different proper ratio of inositol to choline probably due to their specific physiological characteristics. Such beneficial effects of increased nicotinamide levels were confirmed in natural media, molasses and corn starch hydrolyzed sugar media. Meanwhile, such adjustment of inositol to choline ratio could lessen the inhibition of excess inositol on cell growth of the two tested strains in corn starch hydrolyzed sugar media. However, in molasse, such phenomenon was not observed probably since there was higher Ca^2+^ in it. The results indicated that the effects of nutrient factors, such as vitamins, on cell growth and other properties found out from the simple chemically defined minimal medium were an effective measure to use in improving the recipe of natural media at least for baker's yeast.

**Supplementary Information:**

The online version contains supplementary material available at 10.1186/s40643-023-00661-4.

## Introduction

The most widely used raw materials for the production of baker's yeast, also known as *Saccharomyces cerevisiae*, are byproducts of the sugar industry, cane and beet molasses, with considerable variations in composition leading to high variability in yeast manufacturing quality. In addition, with the technological development of the sugar industry, the quality of molasses is becoming worse and less attractive than it has been in the past for yeast production worldwide. Therefore, alternative and inexpensive raw materials are needed to solve the dilemma of high raw material costs (Randez-Gil et al. 1999; Gelinas. 2012; Secches Thaís et al. 2022). Starchy raw materials such as maize and sweet potato are recognized as relatively simple ingredients and promising alternatives to molasses for the production of baker's yeasts. However, using alternative starchy raw materials with the same fermentation process used for baker's yeast production using molasses has always caused problems in industrial production (feedback from collaborating baker's yeast companies). Even when the concentrations and ratios of micronutrients, such as carbon, nitrogen and phosphorus sources, are adjusted during fermentation, the yeast products are usually of worse quality than when using molasses. This negative impact is even more pronounced for baker's yeast products with special properties, e.g., high sugar resistance and high temperature resistance.

Another issue that makes it difficult to identify the critical components in media to baker's yeast performance is the complexity of natural media composition. In this regard, it is necessary and critical to select appropriate media and process conditions for finding the possible components in media that affect baker's yeast performance, which might be important factors hindering the application of starchy raw materials in baker's yeast production. Among the many choices of both laboratory and industrial fermentation media for *S. cerevisiae*, common selections are chemically defined minimal medium (MM) and rich, less-defined yeast peptone dextrose (YPD) medium. Because MM has a known composition and widely used in basic metabolic studies in yeast (Verduyn et al. 1992; Liu et al. 2004; van der Heul et al. 2018; Jakovljević et al. 2019; Knittelfelder et al. 2017; Podpora et al. 2015), it was deemed more suitable and was selected in this study, avoiding the influence of unknown components in other media.

Among the many differences between starchy raw materials and molasses, based on our previous work and the feedback from the collaborating baker's yeast companies as stated before, vitamins were thought to play unignorable roles in the production of baker's yeast when using starchy raw materials. In addition, vitamins are important growth factors for yeast, principally functioning enzymatically and generally acting either as coenzymes or precursors for fully active enzymes, serving a catalytic function in metabolism and directly influencing the growth and corresponding performance of yeast (Perli et al. 2020; Schmidt et al. 2015). It has been reported that an appropriate increase in the levels of nicotinamide, the precursor of NAD^+^, can meet the supply of NAD^+^ required for the intracellular carbon flow biased to the synthesis pathways of substances that promote the tolerance of cells to substances such as glycerol and trehalose; however, excess nicotinamide inhibits the NAD^+^-dependent deacetylase sirtuin (Sir2) in maintaining genomic stability and repairing DNA damage, since nicotinamide is an inhibitor of Sir2 (Anderson et al. 2003; Bitterman et al. 2002). Inositol affects the phosphatidylinositol (PI) content of the yeast cell membrane directly, which contributes to maintaining the tolerance of yeast cells to high concentrations of alcohol (Henderson et al. 2012; Henderson et al. 2014; Ishmayana et al. 2015). However, in contrast to micronutrient studies, few studies have considered the impact of both depleting and supplementing vitamins, leading to vitamins being difficult to control and overlooked during industrial processes, especially within the context of high-quality baker's yeast production using starchy raw materials.

Therefore, the aim of this research was to determine whether and which vitamins are possible critical components in common media for baker's yeast performance and the possible influencing mechanisms. To enable this understanding, common yeast media formulations in both laboratory and industrial settings were compared, specifically the effects of vitamins on growth and the leavening performance of two industrial baker's yeasts with different characteristics in dough fermentation. This work provides meaningful insights into the effects of nicotinamide and inositol on the fermentation performance of two industrial baker's yeasts with different characteristics and offers a new perspective on solving the problem of media substitution in baker's yeast production.

## Materials and methods

### Strains and media

Two industrial *S. cerevisiae* strains were used in this work and were provided by the cooperating company. These two commercial baker’s yeasts were named the high-sugar-tolerant strain (HS) and conventional strain (CS) based on their characteristics and applications.

The yeasts were grown at 30 °C on YPD agar medium (10 g·yeast extract L^−1^, 20 g·tryptone L^−1^, 20 g glucose·L^−1^ and 20 g agar powder L^−1^), and inocula were prepared in YPD. The composition of modified MM* and pretreated and ready-for-use molasses was obtained from the cooperating company, and the detailed corn starch hydrolyzed sugar preparation processes is provided in the Additional file 1.

### Measurement of vitamin and metal elements in media

The contents of different vitamins were determined according to the corresponding Chinese national standards: GB5009.84-2016 for thiamine, GB5009.85-2016 for riboflavin, GB5009.89-2016 for nicotinamide, GB/T18397-2001 for pantothenic acid, GB5009.154-2016 for pyridoxine, GB/T17778-2005 for biotin, and GB5009.270-2016 for inositol. The metal elements were determined according to Chinese national standards GB 5009.268-2016.

### Batch fermentation in shake flasks and bioreactors

A single colony was transferred to fresh YPD in shake flasks at an initial OD_600_ of 0.2 and incubated at 30 °C and 200 rpm for 12 h. After activation twice in YPD, the cells were then collected and washed twice with sterile water and resuspended before inoculation into the fermentation medium. Fermentation was performed in shake flasks with an initial OD_600_ of 0.2 or 1 L bioreactors (Infors Biotechnology, Switzerland) with an initial OD_600_ of 1.5.

### Cell dry weight and viable cell density determination

Measurement of cell dry weight (DCW): A 0.22 μm nitrocellulose filter membrane was placed at 80 °C for 2 h to constant weight. Then, 5–20 mL of fermentation broth was filtered through a dry filter membrane, washed twice with sterile water, and kept at 80 °C for more than 12 h to constant weight. The viable cell density was measured with an Incyte sensor (Hamilton), which enables real-time and online measurement of permittivity correlating with the viable cell density (Feng et al.^2021^).

### Enzyme activity assay

Cell-free extracts were prepared as previously described (Wei et al. 2019) or according to the manufacturer’s protocol. The collected cells were resuspended in disruption buffer with 1 mM PMSF and broken by glass beads (Φ = 0.55 mm) using a FastPrep cell homogenizer (Thermo Savant, Germany). After the samples were centrifuged at 4 °C and 12,000 × g for 10 min (Thermo Fisher Fresco^™^ 17, America), the supernatants were collected as a cell-free extract for the enzyme activity assay.

Immediately after the preparation of cell-free extracts, phosphoenolpyruvate carboxykinase (Pck1) activity was determined according to the PEPCK assay kit (Solarbio Life Sciences, BC3310, China). Sucrase activity was assayed according to the IA Assay Kit (Solarbio Life Sciences, BC0560, China). Trehalose-6-phosphate synthase (TPS) and trehalose-6-phosphate phosphatase (TPP) activity was assayed according to the TPS Assay Kit and TPP Assay Kit (Grace Biotechnology, G0556W, G0582W, China). Superoxide dismutase (SOD) activity was assayed according to the SOD activity detection kit (WST-8 method, Beyotime S0101M). Catalase (CAT) activity was assayed according to the method of the Beyotime catalase detection kit (S0051).

The enzyme activity (U·mg^−1^·protein) was the enzyme activity per milligram of protein. The total cellular protein concentration was measured using the Enhanced BCA protein assay reagent kit (Beyotime Biotechnology, China).

### Determination of leavening power

The leavening power of yeast can be measured by its ability to ferment sugar by the detection of CO_2_ production (Rincón et al. 2001). According to the Chinese national standards, Quality requirements for yeast products-Part 1: Yeast for food processing (GB/T 20886.1-2021), the dough contained 30 g wheat flour, 0.21 g NaCl and a certain amount of sucrose. The doughs containing 4% and 16% sucrose were defined as low-sugar and high-sugar dough, respectively. The sample was washed with sterile water and then mixed with the prepared flour. The fermentation power was evaluated by measuring the CO_2_ production of the dough at 30 °C for 4 h and is presented in milligrams of lost CO_2_ (Stabnikova et al. 2008).

### Quantitative PCR (qPCR) analysis

Total RNA extraction, cDNA preparation, and qRT‒PCR analysis were performed according to the manufacturer's instructions and previous methods (Wei et al. 2019). The primer sequences were as follows (5′–3′): *PNC1*, F: AGACTGGCACAGGATTGTGG, R: ACGGGCCACAAAATACCCTC; *SIR2*, F: ATGGTTCCTTCCCACGTTCC, R: CGTCCAGCCACATTTTTGGG. *ACT1* was used as the reference gene.

### Analysis of intracellular metabolites

The concentrations of glucose, glycerol and ethanol were measured according to Wei et al. (2019). Glutathione (GSH) was assayed according to a GSH and GSSG assay kit (Beyotime Biotechnology, S0053, China). Trehalose was extracted from yeast cells and assayed as described previously (Mahmud et al. 2010) with some modifications. The cells were collected and washed twice with chilled sterile water and resuspended in 1 mL 0.5 M trichloroacetic acid and then broken by a FastPrep cell homogenizer (Thermo Savant, Germany). The absorbance of the supernatant obtained after centrifugation (13,000 rpm, 10 min) was then measured at 630 nm. The intracellular trehalose results are presented in grams of trehalose per gram of dry cells. Intracellular reactive oxygen species (ROS) were detected by a reactive oxygen species assay kit (Solarbio Life Sciences, CA1410, China) according to the manufacturer’s protocol. Intracellular glucose-6-phosphate (G6P) was assayed according to the G6P Assay Kit with WST-8 (Beyotime Biotechnology, S0185, China). NADPH was quantified using an NADP/NADPH Assay Kit (Sigma‒Aldrich, mak312, US). ATP was quantified using an Enhanced ATP assay Kit (Beyotime Biotechnology, S0027, China) according to the manufacturer’s instructions. Uridine diphosphate glucose (UDPG) was extracted with 0.5 mM ammonium acetate and then detected by HPLC (Waters e2695, America) with a UV detector (260 nm) and a Waters Xbridge C_18_ column, mobile phase of 20 mmol·L^−1^ triethylammonium acetate; isocratic elution: 40 min, 1 mL·min^−1^ flow rate, 30 °C column temperature. The phospholipids were extracted using chloroform/methanol (v/v: 2/1) (Bligh and Dyer, 1959), ultrasonic treatment for 10 min and centrifugation at 12,000 × g for 5 min to collect the supernatant. This process was repeated three times. The supernatants containing different classes of phospholipids were separated on untreated silica plates by thin layer chromatography, and the developing solvent was chloroform/methanol/H_2_O/triethylamine (50:40:8:50, by volume). Then, each separated phospholipid was converted to fatty acid methyl esters by acid-catalyzed methyl esterification for quantification by gas chromatography (GC) with a flame ionization detector (FID) (Agilent 7890B, America) and a DB-23 column (30 m × 0.32 mm × 0.25 μm, Agilent) (Liu et al. 2015).

## Results and discussion

### Analysis of yeast strain characteristics

**Fig. 1:**
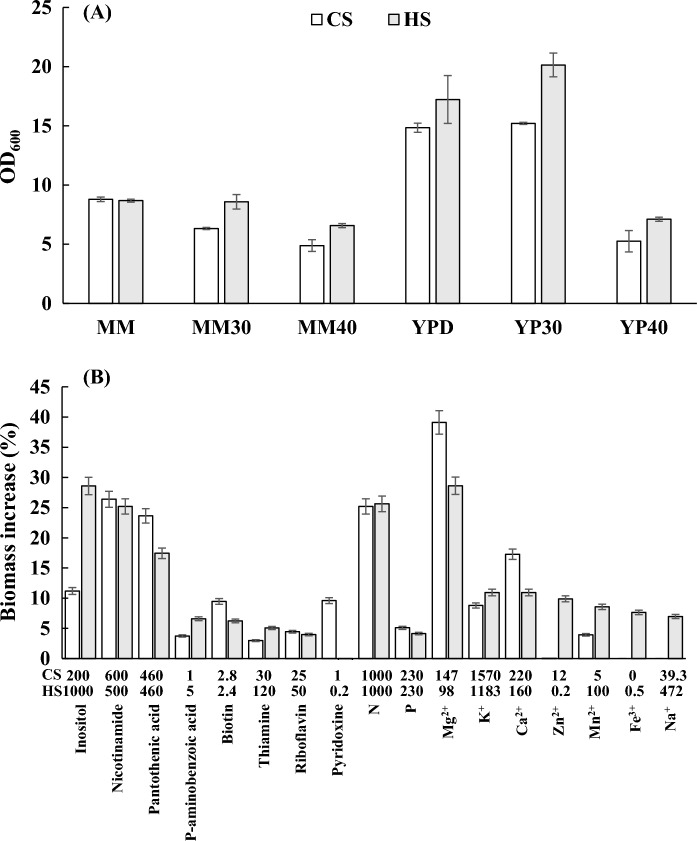
Growth in MM and YPD with different glucose contents **A** and the effects of different nutritional ingredients on the growth **B** in MM of the two industrial strains. The data in the table below the graph represent the optimized concentration of the corresponding nutrient factors. MM, YPD: 2% glucose; MM30, YP30: 30% glucose; MM40, YP40: 40% glucose

Compared to CS, HS should be more suitable for leavening doughs with a high sugar content, as defined in Chinese national standards (GB/T 20886.1-2021). To verify the different characteristics, the growth as well as the related fermentation performance of the two baker's yeast strains was investigated in MM and YPD media with different sugar concentrations in flasks. As shown in Fig. 1A, when the concentration of glucose in the media increased to 30% and 40%, the growth advantage of HS became increasingly apparent compared to CS. In addition, as shown in Fig. 5A, the leavening power of CS was better than that of HS in low-sugar dough (dough with 4% sugar content), while in high-sugar dough (dough with 16% sugar content), HS showed a higher leavening power than CS. These results indicated the better tolerance of HS to high sugar, which was consistent with the performance characteristics of the two strains as described by the company.

### Differences in nutritional composition between various media

The differences in vitamin compositions were explored in various media, including sugarcane molasses, the common raw material for production at local yeast enterprises in Shandong Province China; corn starch hydrolyzed sugar, a potential substitute material for molasses; YPD medium, the common medium used in laboratories; and MM, synthetic complete medium. It was obvious that there were large differences in vitamin composition among the four media, especially for nicotinamide, biotin, inositol and pantothenic acid between corn starch hydrolyzed sugar and sugarcane molasses medium, as shown in Table 1. The content of most vitamin components in corn starch hydrolyzed sugar was lower than that in sugarcane molasses medium, but the inositol content was much higher. In YPD, a nutrient-rich medium commonly used in the laboratory, pantothenic acid levels were exceptionally high compared to that in the other two natural media. Additionally, the content of metallic elements in different media also varied considerably.

**Table 1:** Differences in vitamin content between various media

Component (mg·L^−1^)	MM	YPD	Molasses	Hydrolyzed sugar
Pyridoxine	0.4	ND	1.1	0.06
Nicotinamide	0.4	8.9	38.1	0.05
Pantothenic acid	0.2	1481.7	16.0	0.04
Inositol	2.0	1530.0	2552.7	3525.9
Riboflavin	0.2	1.1	2.4	ND
Thiamine	0.4	0.129	3.3	ND
Biotin	0.002	ND	69.8	0.55
P-aminobenzoic acid	0.2	0.417	ND	ND
Mg	48.8	29.2	456.0	27.48
Ca	27.2	0.9	260.2	1.58
Zn	0.02	2.3	0.6	0.08
K	293.5	416.0	453.0	31.32
Na	39.3	609.5	1.2	ND
Cu	0.003	ND	ND	0.016
Fe	–	1.0	5.5	0.058
Mn	–	0.9	0.9	0.05
Cd	–	0.006	0.004	ND
Pb	–	0.009	0.001	0.002
As	–	ND	0.006	ND
Se	–	ND	0.001	ND

“ND” indicates no detection. The concentration of all components in molasses was re-calculated at fermentable sugars of 2%

### Effects of different nutritional ingredients on the growth of the two yeast strains

As shown in Table 1, the range of concentrations of each nutrient factor varies considerably in different media. The effect of each nutritional ingredient on the growth of the two yeast strains was investigated in flasks, and the results are shown in Fig. 1B. Three vitamins, nicotinamide, pantothenic acid and inositol, enhanced the growth of both yeast strains by more than 10%. When the concentration of pantothenic acid was increased to 460 mg·L^−1^, the growth of CS and HS was increased by 24.4% and 17.4%, respectively. The concentrations of nicotinamide and pantothenic acid in the medium at this point were much higher than their levels in the media displayed in Table 1, suggesting that supplementation of the two vitamins in the production media may further increase the final yeast yield. The growth of CS and HS was increased by 11.2% and 28.6% when the inositol concentration in the medium was 200 mg·L^−1^ and 1000 mg·L^−1^, respectively. However, both inositol concentrations were much lower than the actual inositol levels in the production media in Table 1, and it is worth further exploring how to reduce the negative effects of excessive inositol on growth. For the metal elements tested, Mg^2+^ had the most noticeable effect on the increase in growth of the two strains. These results demonstrate that for the production of baker's yeast, in addition to metallic elements such as Mg^2+^ that have received much attention during industrial processes, the role of vitamins is so important that it should be given more attention, especially when using starchy raw materials, whose vitamin composition is lower than that of molasses.

### Effects of nicotinamide and inositol supplementation on the growth of the two yeast strains

**Fig. 2:**
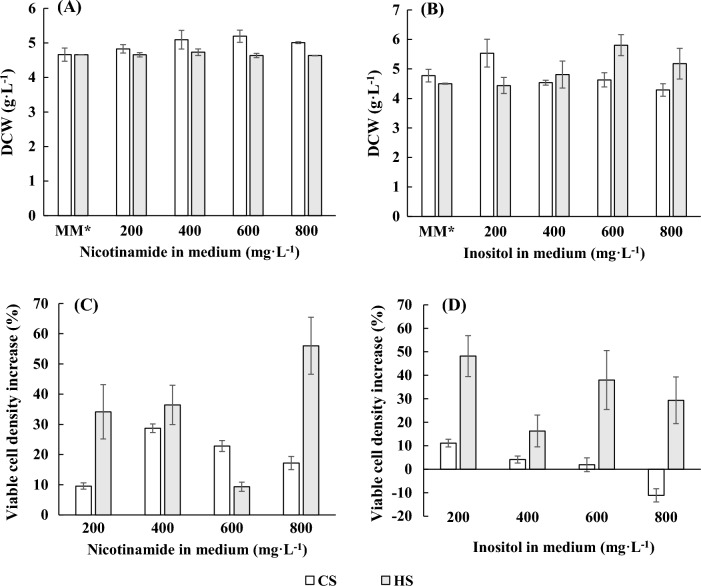
Effects of nicotinamide and inositol supplementation on the growth of the two yeast strains. DCW of the two strains **A**, **B**; effects of nicotinamide **C** and inositol **D** supplementation on the viable cell density of CS and HS

After nitrogen source supplementation to a concentration of 1000 mg·L^−1^, the growth of both strains was increased by approximately 25%, as shown in Fig. 1B. This result suggested that the nitrogen source in MM was at a low level. Therefore, the nitrogen in MM was optimized to a proper carbon-to-nitrogen (C/N) ratio for yeast in high-density cultivation, and the modified medium is noted as MM* in the following studies. A fermentation process with an exponential feeding model was developed for the control of media flow addition in bioreactors (Additional file 1, 2). Using the fermentation process established in bioreactors and the modified MM* described above, studies on the effects of supplementation of two vitamins, nicotinamide and inositol, were carried out, and the effects on the growth of the two yeast strains are shown in Fig. 2.

Nicotinamide had a more pronounced promotion effect on the growth and number of viable cells of the two strains. For CS, the maximum biomass was reached at an increased nicotinamide concentration of 600 mg·L^−1^. At a nicotinamide concentration of 400 mg·L^−1^, CS had the most viable cells, with an increase of 28.07% compared to the control (Fig. 2A, C). For HS, the addition of nicotinamide had no significant effect on the total biomass, but at a concentration of 800 mg·L^−1^, the viable cells reached a maximum level, with an increase of approximately 56.02% compared to the control (Fig. 2A, C). After inositol supplementation at a concentration of 200 mg·L^−1^, both the total biomass and number of viable cells of CS increased obviously (Fig. 2B, D). The maximum total biomass of HS was reached at an inositol level of 600 mg·L^−1^, and the number of viable cells increased the most at an inositol concentration of 200 mg·L^−1^, an increase of 46.99% (Fig. 2B, D).

**Fig. 3:**
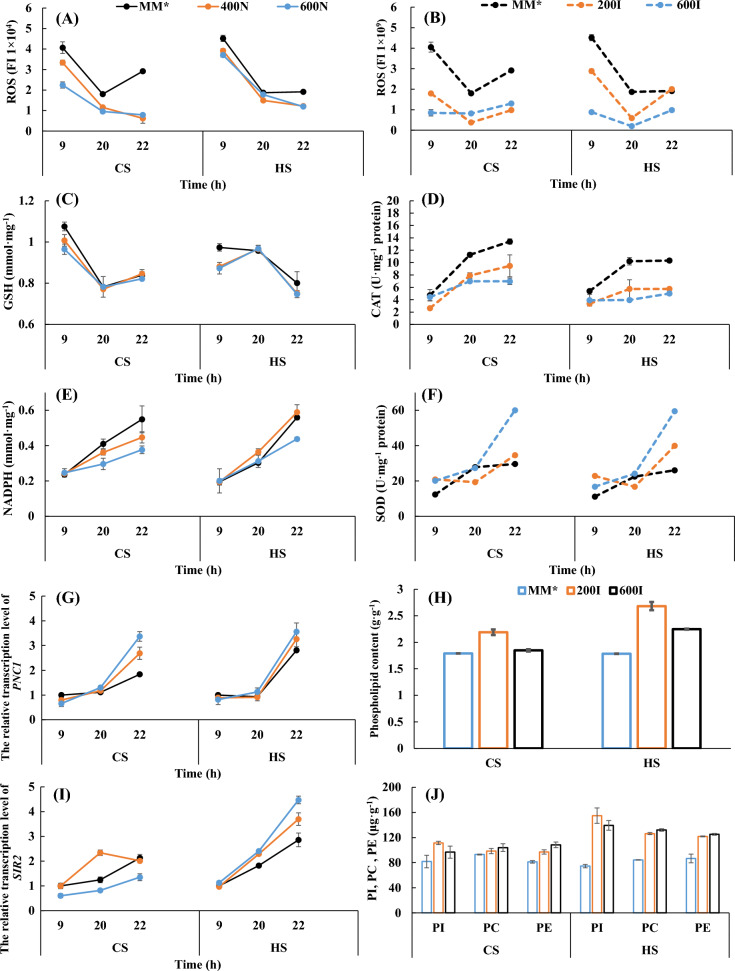
Effects of nicotinamide supplementation on ROS, GSH, NADPH, *SIR2*, *PNC1* (**A**, **C**, **E**, **G**, **I**) and inositol supplementation on the ROS, CAT, SOD and phospholipid contents (**B**, **D**, **F**, **H**, **J**) of the two strains. 400N and 600N: 400 mg·L^−1^ and 600 mg·L^−1^ nicotinamide in MM*; 200I and 600I: 200 mg·L^−1^ and 600 mg·L^−1^ inositol in MM*. *PI* phosphatidylinositol, *PC* phosphatidylcholine, *PE* phosphatidylethanolamine

To obtain a preliminary understanding of how nicotinamide and inositol supplementation affects cell growth and cell activity, we further investigated the changes in some metabolites as well as changes in the transcription of two genes. The free radical theory of aging suggests that oxidative damage caused by intracellular ROS production is the ultimate cause of cellular senescence and potential death and a major determinant of cellular lifespan, and intracellular ROS levels are one of the most important influences leading to cell death (Sauve et al. 2006). The higher the mitochondrial activity is, the more susceptible the cell is to environmental stimuli to produce more ROS, leading to oxidative damage to genes and proteins and shortening the lifespan of the cells. For both baker's yeast strains, the intracellular ROS content was essentially lower than that of the control without nicotinamide or inositol supplementation throughout the fermentation cycle. Compared to the control, the intracellular ROS content of CS changed most significantly at the logarithmic growth phase (9 h data) after nicotinamide supplementation, with a reduction of 44.8% at a nicotinamide concentration of 600 mg·L^−1^ (Fig. 3A). For HS, intracellular ROS levels were also reduced after nicotinamide supplementation (Fig. 3B). The reduction in intracellular ROS levels was less pronounced in HS after inositol supplementation than in CS.

Moreover, reducing substances such as GSH, NADPH, and SOD are usually produced intracellularly to eliminate ROS to resist oxidative stress and protect cells from ROS damage (Liu et al. 2022; Xu et al. 2018). As shown in Fig. 3C, E, supplementation with nicotinamide resulted in lower GSH and NADPH for the two strains, which might be the consequence of the decreased ROS levels, accompanied by a drop in demand for ROS-eliminating reducing substances. In contrast to nicotinamide, after inositol supplementation, SOD assumed a major role in ROS elimination in both strains, as SOD enzyme activity increased obviously (Fig. 3F). However, the enzyme activity of CAT decreased instead (Fig. 3D). In addition to CAT, Itr2p, an inositol transporter, also has the ability to scavenge hydrogen peroxide. Itr2p is active when the other transporter Itr1p is inhibited under high levels of inositol outside the cells (Santos et al. 2019). Thus, the increased level of Itr2p in cells may be one of the causes of the reduced need for CAT to clear hydrogen peroxide. These results indicated that nicotinamide as well as inositol supplementation might contribute to in vivo ROS scavenging, thus protecting cells from ROS damage.

As stated before, nicotinamide is a precursor of NAD^+^ and an inhibitor of NAD^+^-dependent Sir2 (Anderson et al. 2003; Bitterman et al. 2002), and too much nicotinamide inhibits Sir2 from maintaining genomic stability and repairing DNA damage. Similar to the *SIR2* gene, *PNC1* is another important gene associated with cellular lifespan, and the expression of both is influenced by nicotinamide (Lin et al. 2000; Sandmeie et al. 2002). *PNC1* encodes a nicotinamidase that catalyzes the deamidation of nicotinamide and can convert nicotinamide to nicotinic acid in the remedial synthesis pathway of NAD^+^, stabilizing the concentration of nicotinamide in cells, and the expression of its encoding gene, *PNC1*, is influenced by the intracellular nicotinamide content (Sandmeier et al. 2002). After nicotinamide supplementation, the transcript levels of both intracellular *SIR2* (except in CS) and *PNC1* of the two strains were increased in late fermentation, as shown in Fig. 3G, I, suggesting the positive effects of a certain content of nicotinamide on the cellular lifespan and cell growth.

As a component of PI, inositol in the medium directly influences the phospholipid composition, especially the PI content of the yeast cell membrane. PI serves as a precursor for various signal transduction molecules, and a lack of PI can reduce cell viability (Xia et al. 2011; Suliman et al. 2022), which makes PI an essential phospholipid in *S. cerevisiae* (Guo et al. 2018). As shown in Fig. 3H, for both strains, the PI contents increased obviously after inositol supplementation, which might be beneficial for higher cell viability.

### Proper nicotinamide and inositol contents enhanced trehalose synthesis in the two yeast strains

Trehalose acts as a major intracellular compatible solute and high osmolarity protector, enhancing yeast tolerance to high sugar concentrations (Shima and Takagi 2009). In addition, trehalose is a typical metabolite recognized to extend the shelf life of yeast. Therefore, enhancing trehalose synthesis is an effective way to improve the high-sugar tolerance and shelf life of yeast.

**Fig. 4:**
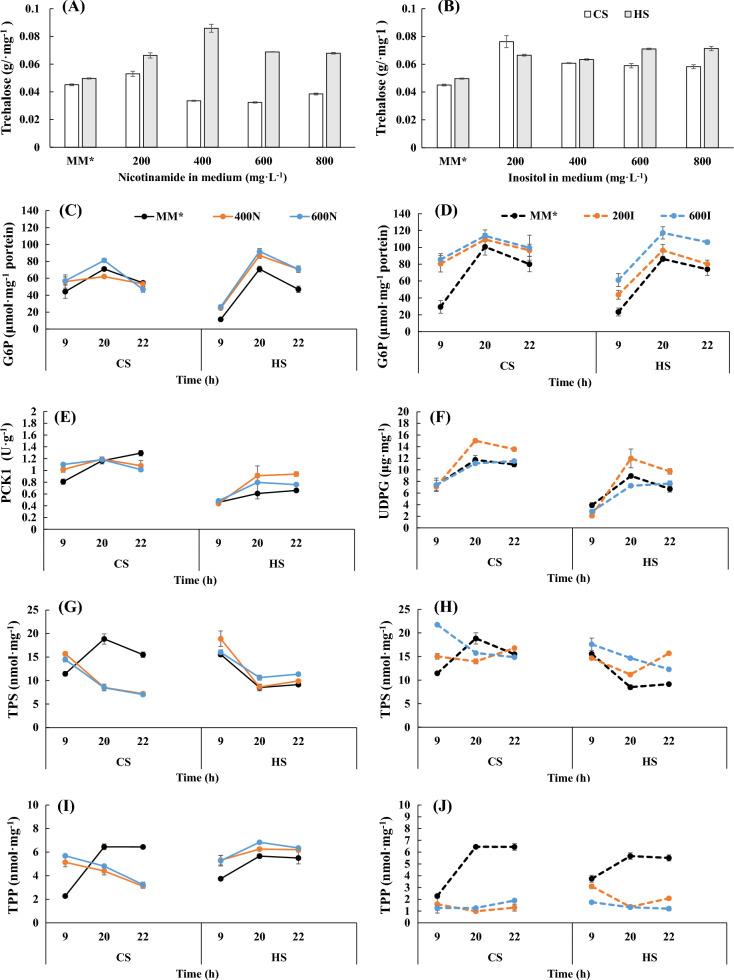
Effects of nicotinamide and inositol supplementation on trehalose synthesis in the two strains. 400N and 600N: 400 mg·L^−1^ and 600 mg·L^−1^ nicotinamide in MM*; 200I and 600I: 200 mg·L^−1^ and 600 mg·L^−1^ inositol in MM*

During yeast trehalose synthesis, TPS converts UDPG and G6P to trehalose-6-phosphate, and subsequently, TPP converts trehalose-6-phosphate to trehalose. PCK1 is a central molecule regulating glycolysis, the TCA cycle and gluconeogenesis and plays an important role in the metabolic pathways of yeast. As shown in Fig. 4A, C, E, G, I, for HS, after nicotinamide supplementation, the trehalose synthesis pathway was enhanced, as evidenced by the increased content of G6P and the key enzymes of trehalose synthesis, including PCK1, TPS and TPP; however, unlike HS, when the nicotinamide concentration was above 400 mg·L^−1^, the trehalose synthesis pathway of CS was weakened. After inositol supplementation, the trehalose synthesis pathway of the two strains was also enhanced (Fig. 4B); in particular, the contents of G6P and UDPG were increased (Fig. 4D, F). In addition, after inositol supplementation, the TPS activity of HS increased, but for CS, the TPS activity was only higher than that of the control in the logarithmic growth phase, and the TPP enzyme activity of the two strains decreased, as shown in Fig. 4H, J. These results indicated that the substrate content may be the key factor limiting the synthesis of trehalose rather than the enzyme activity. Thus, supplementation with both nicotinamide and inositol in appropriate amounts enhances the accumulation of trehalose in both baker's yeast strains.

### Effect of nicotinamide and inositol supplementation on the leavening power of the two yeast strains

**Fig. 5:**
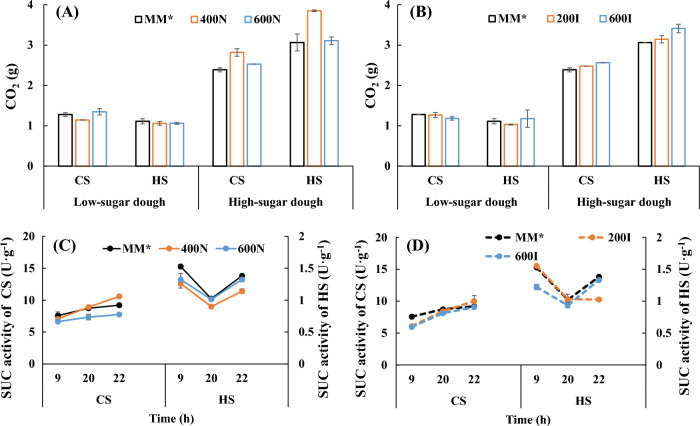
Effects of nicotinamide and inositol supplementation on the leavening power (**A**, **B**) and sucrase (SUC) activity (**C**, **D**) of the two strains

A crucial biotechnological feature of the bakery yeast end product is its fermentative or dough-raising power. This property depends on many factors, such as sucrase activity and osmotolerance (Zhang et al. 2016). In this study, we explored the sucrase level and the leavening ability of baker’s yeast in dough with different sugar contents after nicotinamide and inositol supplementation. Comparing the sucrose content of the two yeast strains reveals that CS secretes much more sucrase than HS (Fig. 5C, D), which is an important reason why HS has better high-sugar tolerance than CS.

As shown in Fig. 5A, B, in low-sugar dough, after both nicotinamide supplementation and inositol supplementation, the leavening ability of both strains did not increase significantly but tended to decrease slightly. In high-sugar dough, at a nicotinamide concentration of 400 mg·L^−1^, the leavening ability of both strains was obviously enhanced compared to the control in Fig. 5A; at this time, the trend of sucrase in the two yeast strains was opposite to the leavening ability (Fig. 5C), consistent with results indicating that sucrase activity negatively affected the leavening ability of a baker’s yeast strain under high-sucrose conditions (Zhang et al. 2016). However, the changes in sucrase activity after inositol supplementation were slightly different. At the end of fermentation, except at 200 mg·L^−1^ inositol addition, sucrase activity remained largely unchanged (Fig. 5D).

As previously discussed, after inositol supplementation, the phospholipid compositions changed obviously, namely, the contents of phosphatidyl choline (PC) and phosphatidyl ethanolamine (PE) (Fig. 3J). Due to differences in the characteristics of PC and PE, PC and PE are more likely to form bilayer and nonbilayer phospholipids (Cullis and De Kruijff, 1979). Thus, the changes in PE/PC ratios will influence the nature of the membrane. It is possible that changes in membrane properties will influence the activities of certain membrane proteins and further influence the high concentration sugar tolerance of yeast cells (Chi et al. 1999; Ishmayana et al. 2015).

### Positive effects of nicotinamide supplementation and choline addition on the growth and leavening power of the two strains in natural media

**Fig. 6:**
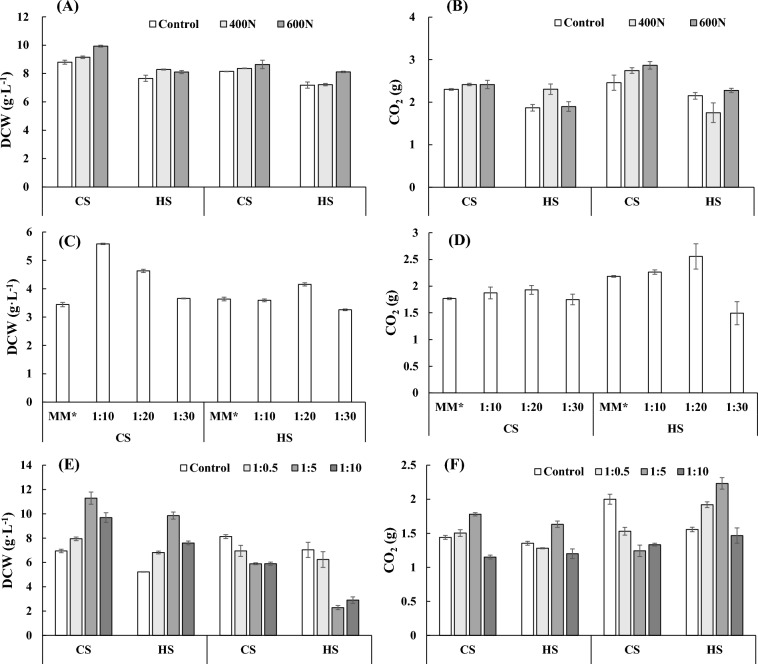
Effects of nicotinamide supplementation and choline addition on the growth (**A**, **C**, **E**) and leavening power (**B**, **D**, **F**) of the two strains in molasses and corn starch hydrolyzed sugar

Although the positive effects of nicotinamide and inositol on the production and product performance of the two baker's yeasts have been verified before, there are still problems in natural media. As previously stated, the natural medium composition is very complex and contains many substances of unknown composition, which leads to many unknown problems when optimizing and replacing raw materials. Here, the effect of nicotinamide adjustment was tested in natural media. The addition of nicotinamide to the medium increased the dry weight of both baker's yeast strains (Fig. 6A), and the promotion effect on cell growth was more significant in corn starch hydrolyzed sugar media than molasses. The leavening power of CS in low-sugar dough and the leavening power of HS in high-sugar dough were determined. As shown in Fig. 6B, the addition of nicotinamide could promote the leavening power in corn starch hydrolyzed sugar media, while in molasses, the increase in the leavening power of CS was more obvious. Overall, the supplementation of nicotinamide in the two media was beneficial for both yeast strains.

In addition, it should be noted that compared to the deficiency of a certain nutrient, it is much harder to alleviate the problem of excessive content of nutrient elements that are difficult to remove. In fact, the amount of inositol in natural medium is much higher than the actual requirement (Table 1). Consideration needs to be given to how to reduce or even release the inhibition of excessive inositol. Choline is a PC precursor, and the intracellular PC content increased obviously under the simultaneous presence of choline and inositol in media (Gaspar et al. 2006). The increase in PC, favorable to improving phosphatidic acid synthesis, would consequently alleviate the transcriptional repression of the UAS_INO_ element caused by ectopic inositol-mediated blocker protein Opi1p due to the insufficient intermediate phosphatidic acid (Carman and Han 2011). Therefore, the effect of choline in reducing the inhibition of excessive inositol on growth and leavening power in molasses and corn starch hydrolyzed sugar media was investigated.

The positive effects of choline addition on the cell growth and leavening power of both strains were verified in MM*, as shown in Fig. 6C, D. The optimum ratio of inositol to choline varied for the two strains, 1:10 for CS and 1:20 for HS. Considering the much higher content of inositol in the natural medium, the ratio of inositol to choline was adjusted down to a minimum of 1:10. In the corn starch hydrolyzed sugar medium, when the ratio of inositol to choline was 1:5, the growth and leavening power of both strains was promoted (Fig. 6E, F). However, the growth of both strains was severely inhibited when choline was added to the molasses. This strong inhibition of growth neutralized the promotion in leavening power for HS when the ratio of inositol to choline was 1:05 and 1:5 (Fig. 6E, F). This phenomenon is most likely due to differences in the composition of the two natural media. Compared to corn starch hydrolyzed sugar media, molasses contains many more metal ions, such as Ca^2+^ and Mg^2+^ (Table 1). The high Ca^2+^ concentration inhibits the activity of Ca^2+^-ATPase in the membrane rich in PC (Nitsche et al. 2018), resulting in slower export of Ca^2+^ from the cytosol in yeast. In addition, the increasing PC in the membrane after the prerequisite choline addition exacerbates the problem of Ca^2+^ export since PC binds to Ca^2+^ more weakly than other phospholipid components in the membrane, namely, phosphatidylserine and phosphatidylglycerol (Sinn et al. 2006). The above problems of Ca^2+^ export from the cytosol under high Ca^2+^ concentrations would properly lead to an imbalance of intracellular Ca^2+^ and consequently the unhealthy growth of the yeast cells. Similar to metal ions, there was also an immense difference in amino acid content between the molasses (nearly 500 mM) and the corn starch hydrolyzed sugar media (hardly any) used in this study. In molasses, the amino acids can easily react with the added choline and form new components called choline amino acids, also known as ionic liquids (Sivapragasam et al. 2019). Due to the high amount of amino acids and added choline in molasses, the newly formed choline amino acids can easily reach the minimum inhibitory concentration and minimum bactericidal concentration reported for different microorganisms, thus resulting in choline addition inhibiting cell growth (Sivapragasam et al. 2019). The complex composition of molasses limits the application of the existing research findings based on synthetic media. There are still many mechanisms that remain unclear and hinder the development of yeast production media.

## Conclusion

In the current work, we presented meaningful aspects of nicotinamide and inositol supplementation, confirming their positive effects on the production and product performance of two industrial baker's yeasts with different characteristics by reducing ROS, enhancing trehalose synthesis, and rearranging the phospholipid composition. The positive effects of the two vitamins were also confirmed in natural media. In addition, choline was found to be a suitable choice to reduce high-inositol-content inhibition in corn starch hydrolyzed sugar media. The results suggest the necessity of proportioning vitamins in media for baker's yeast production.

### Supplementary Information

**Additional file 1. S1. **The composition of modified MM*.  **S2.** The detailed preparation processing of corn starch hydrolyzed sugar.  S3. Establishment of the fermentation process and MM composition modification.

**Additional file 2. Fig. S1 **Adjustment of C/N ratio of MM in bioreactors.

## Data Availability

All data analyzed during this study are included in this article.
