# Supplementary material for: The impacts of nicotinamide and inositol on the available cells and product performance of industrial baker's yeasts
Source: Bioresour Bioprocess. 2023 Jul 22;10(1):41. doi: 10.1186/s40643-023-00661-4 (PMC10991249; doi:10.1186/s40643-023-00661-4)
Supplement: Supplementary file 1 — Additional file 1. S1. The composition of modified MM*. S2. The detailed preparation processing of corn starch hydrolyzed sugar. S3. Establishment of the fermentation process and MM composition modification. [file 40643_2023_661_MOESM1_ESM.doc]

**S1. The composition of modified MM***

**The composition of modified MM***

The modified MM* consisted of: 1 g (NH_4_)_2_SO_4_ ·L^-1^, 0.125 g K_2_HPO_4_ ·L^-1^, 0.875 g KH_2_PO_4_ ·L^-1^, 0.5 g MgSO_4_ ·L^-1^, 0.1 g CaCl₂ ·L^-1^, 0.1 g NaCl ·L^-1^, sterilized at 115 ℃ for 30 min. After filtration sterilization, potassium iodide was added to 0.1 mg·L^-1^, three trace elements were added: 0.07 mg ZnSO_4_ ·L^-1^, 0.01 mg CuSO₄ ·L^-1^, 0.05 mg CaCl₂ ·L^-1^ phosphate was added to 0.01 mL·L^-1^. Eight vitamins were added: 0.4 mg nicotinamide ·L^-1^, 2 mg inositol ·L^-1^, 0.2 mg P-aminobenzoic acid ·L^-1^, 0.2 mg pantothenic acid ·L^-1^, 0.4 mg thiamine ·L^-1^, 0.2 mg riboflavin ·L^-1^, 0.4 mg pyridoxine ·L^-1^ and 0.002 mg biotin ·L^-1^. Glucose was added to the final concentration of 20 g·L^-1^, pH 5.5.

**S2. The detailed preparation processing of corn starch hydrolyzed sugar**

The corn was placed in a 60ºC drying oven and baked for 24 hours to remove excess moisture. The dried corn was crushed and passed through a 200-mesh sieve to eliminate coarse impurities like corn bran. After 0.1% CaCl_2_ (corn flour basis) addition, the pH of the slurry (250 g corn flour ·L^-1^) was adjusted to 5.6, and 40 U·g^-1^ corn flour high-temperature α-amylase (provided by Shandong KDN Biotechnology Co., Ltd.) was added into the slurry. The mixture was incubated at 90ºC until the iodine test showed reddish-brown color. Then the pH of the mixture was adjusted to 4.6 before glucoamylase addition (100 U·g^-1^ corn flour, provided by Shandong KDN Biotechnology Co., Ltd.). The mixture was then incubated at 70ºC until the iodine test showed the original color, indicating the completion of hydrolysis. After centrifugation (5000 rpm, 10 min), the supernatant was filtered to obtain the corn starch hydrolyzed sugar solution.

**S3. Establishment of the fermentation process and MM composition modification**

The final products are cells with high activity during baker’s yeast production. Due to the Crabtree effect, yeast perform aerobic fermentation of glucose to ethanol in glucose- and oxygen-rich environments rather than the expected metabolism of aerobic respiration. Therefore, it is necessary to control the sugar concentration in the medium to avoid a decrease in yeast biomass yield. To achieve this, an exponential feeding model was developed for the control of media flow addition during fermentation. With the exponential feeding model, the real-time glucose concentration in the medium was under precision control, thus ensuring that the ethanol concentration in the medium was not higher than 3 g·L^-1^ during the whole fermentation process. The exponential feeding model was applied in the following studies.

In addition to the feeding mode of high-density cultivation of baker's yeast, another issue to be considered is the carbon-to-nitrogen (C/N) ratio in the medium, which is considered to be a crucial parameter in yeast production processess (Danesi et al. 2006; Manikandan K and Viruthagiri, 2010). Studies have shown that the optimal C/N ratio is approximately 10 (Danesi et al., 2006). However, the C/N ratio in the MM was nearly 40, indicating a massive lack of N sources. Therefore, to avoid covering up the real phenomenon due to the lack of N sources when investigating the effects of vitamins on yeast growth and fermentation performance, the N in MM was supplemented and adjusted in this section. As shown in Fig. S1, when the optimized N source was 1000 mg·L^-1^ for both strains, the C/N ratio was 8. The modified medium with the increased N concentration is noted as MM* in the following studies.

### References

Danesi E D G, Miguel Â S M, Rangel-Yagui C d O, de Carvalho J C M, Pessoa A (2006) Effect of carbon:nitrogen ratio (C:N) and substrate source on glucose-6-phosphate dehydrogenase (G6PDH) production by recombinant *Saccharomyces cerevisiae*. J Food Eng 75: 96-103.

Manikandan K, Viruthagiri T (2010) Optimization of C/N ratio of the medium and fermentation conditions of ethanol production from Tapioca starch using co-culture of *Aspergillus niger* and *Saccharomyces cerevisiae*. Int J ChemTech Res 2:947-55.
