# Supplementary figures and images for: The impacts of nicotinamide and inositol on the available cells and product performance of industrial baker's yeasts
Source: Bioresour Bioprocess. 2023 Jul 22;10(1):41. doi: 10.1186/s40643-023-00661-4 (PMC10991249; doi:10.1186/s40643-023-00661-4)

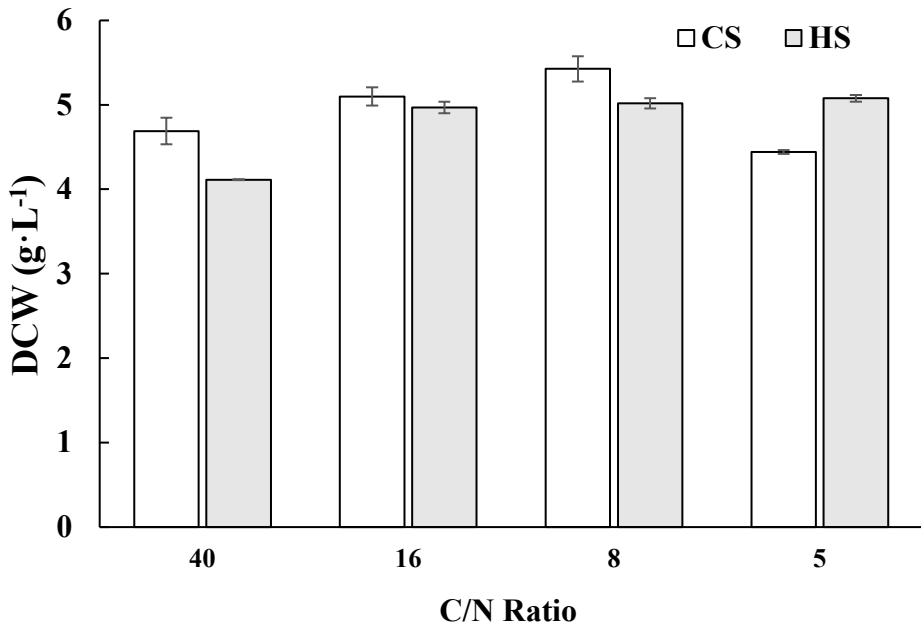

Supplement: Supplementary file 2 — Additional file 2. Fig. S1 Adjustment of C/N ratio of MM in bioreactors. [file 40643_2023_661_MOESM2_ESM.pdf]
